# Continual rehabilitation motivation of patients with postparalytic facial nerve syndrome

**DOI:** 10.1007/s00405-021-06895-2

**Published:** 2021-05-24

**Authors:** Maike Osthues, Anna-Maria Kuttenreich, Gerd Fabian Volk, Christian Dobel, Bernhard Strauss, Uwe Altmann, Orlando Guntinas-Lichius

**Affiliations:** 1 Department of Otorhinolaryngology, Jena University Hospital, Friedrich-Schiller University Jena, Am Klinikum 1, 07747 Jena, Germany; 2 Facial Nerve Center Jena, Jena University Hospital, Friedrich-Schiller University Jena, Am Klinikum 1, 07747 Jena, Germany; 3 Institute of Psychosocial Medicine, Psychotherapy and Psycho-Oncology, Jena University Hospital, Jena, Germany

**Keywords:** Rehabilitation, Motivation, Facial nerve, Chronic facial palsy, Facial paralysis, Anxiety, Depression, Patient reported outcome measure, Quality of life

## Abstract

**Purpose:**

To evaluate the continued rehabilitation motivation in patients with postparalytic facial synkinesis (PFS).

**Methods:**

In this single-center cross-sectional survey, the multidimensional patient questionnaire for assessment of rehabilitation motivation (PAREMO-20) was used to assess the rehabilitation motivation. Associations Sunnybrook and Stennert index grading, Facial Clinimetric Evaluation (FaCE) survey, general quality of life (SF-36), Liebowitz Social Anxiety Scale (LSAS), Patient Health Questionnaire (PHQ)-9, technology commitment and affinity, and interest in further therapy were analyzed.

**Results:**

69 adults with PFS (73% women; median age: 54 years) answered the survey. In comparison to prior treatment forms, there was a significant higher future interest in computer-based home facial training (*p* < 0.0001). For PAREMO Psychological burden subscore, SF36 Emotional role was the highest negative correlative factor (*p* < 0.0001). For PAREMO Physical burden subscore, SF-36 General health was the highest negative correlative factor (*p* = 0.018). Working (*p* = 0.033) and permanent relationship (*p* = 0.029) were the only independent factors correlated to PAREMO Social Support Subscore. Higher positive impacts of technology affinity was inversely correlated to PAREMO Knowledge subscore (*p* = 0.017). Lower SF-36 Role physical subscore *p* = 0.045) and a lower SF-36 General health (*p* = 0.013) were correlated to a higher PAREMO Skepticism subscore.

**Conclusions:**

Patients with PFS seem to have a high facial motor and non-motor psychosocial impairment even after several facial therapies. Rehabilitation-related motivation increases with both, higher facial motor and non-motor dysfunction. Social and emotional dysfunction are drivers to be interested in innovative digital therapy forms.

**Supplementary Information:**

The online version contains supplementary material available at 10.1007/s00405-021-06895-2.

## Introduction

Severe facial nerve paralysis can lead in the chronic phase to altered patterns of muscle contraction and postparetic facial synkinesis (PFS). PFS is a disfiguring condition characterized by involuntary contraction of one or more facial muscles during voluntary movement of other muscles [1, 2]. Diminished facial expression, especially the inability to smile and affected face-to-face communication are the major non-motoric disabilities [3]. Altered facial motor function and the non-motor psychosocial problems can severely decrease quality of life in facial palsy patients [4]. Many patients with PFS are never referred to a specialist or with a too long delay to receive a treatment [5].

The patient’s perspective on the demand of and access to therapy for PFS has also be considered. An effective rehabilitation is linked to the patient’s motivation and compliance regarding the rehabilitation process [6, 7]. Physical rehabilitation therapy is the most often prescribed measure [8]. However, access to specific facial rehabilitation is limited. Complementary, patients with PFS are advised to carry out self-reliant home-based exercise programs [9]. However, these physiotherapeutic exercises are often performed incorrectly, not as frequently as recommended, or are stopped after a certain time [10]. Digital technology with web-based surveillance of the patients at home by facial therapy specialists could potentially improve the long-term continual access to therapy [11].

Recently, we performed a large cross-sectional survey to study the acceptance of emotion-sensitive training systems for patients with facial palsy. First results are published elsewhere [12]. The present study on the subgroup of patients with PFS was performed for better understanding the long-term rehabilitation motivation, expectations and its influencing factors. Patients with PFS were invited to complete a survey including validated patient-reported outcome measures on their rehabilitation motivation, motor and non-motoric facial dysfunction, quality of life, and technology affinity. We hypothesized that patients with higher motoric and non-motoric dysfunctions have a higher and continuing motivation for long-term rehabilitation.

## Material and methods

### Study design and inclusion criteria

This study was part of a cross-sectional survey of adult patients with facial palsy (International Statistical Classification of Diseases and Related Health Problems, German Modification (ICD-10-GM) code: G51.0). These patients had visited the Department of Otolaryngology, Jena University Hospital, Germany, between 2006 and 2016 and had given written consent to be contacted of research purposes. 300 patients counting backwards from last contact with the department were contacted by post or directly in the department (Flowchart, see Supplement Fig. 1). 81 patients (response rate: 27%) answered the questionnaire. The present study analyzed the data of the patients with (PFS) at the time of the survey. The criteria were as follows: (a) a unilateral peripheral facial palsy; (b) incomplete recovery, (c) interval between onset and assessment at least 6 months; (d) at least one facial electromyography (EMG) confirming a PFS including synkinetic activity between periocular and perioral facial muscles [1, 13]. 204 of the contacted 300 patients fulfilled these PFS criteria (69 answered, 135 did not answer; response rate of PFS patients: 33.8%). Hence, these 69 patients with PFS constituted the opportunity sample. All facial palsy-related data were prospectively collected in the department and were complete for all 69 patients. The institutional review board of the Jena University Hospital approved the cross-sectional survey and the study protocol for the additional retrospective data analysis.

### Assessment with several patient-reported outcome measures

Socio-demographic data were collected. Furthermore, questions were asked about initial treatment during the acute phase of the disease, prior treatment in the chronic phase of the disease and current most burden complaints. A list of therapy options was offered with the question to select therapy forms the patient would use like to continue or in be interested in. This list included standard therapy forms (Home mirror training, Facial training with therapist, biofeedback training, facial electrostimulation, acupuncture, light/heat/cold therapy), drugs (supportive eye protection with drops/ointment, botulinum toxin), surgery (nerve surgery, eye lid surgery, angle of the mouth surgery), and also the newer technology allowing a computer-based home training [14]. Finally, we offered the implantation of a potentially in the future available facial pace maker [15]. All therapy forms were shortly explained. User preferences regarding a described concept of an emotion-sensitive training system and sleep quality of the patients were also recorded and published elsewhere [12]. The survey covered several validated patient-reported outcome measures (PROMs). The patient questionnaire for assessment of rehabilitation motivation (PAREMO-20) was used as primary outcome parameter. PAREMO-20 is a multidimensional instrument to determine the general rehabilitation-related motivation of the patients [16, 17]. PAREMO-20 consists of 20 items forming six subscales: “psychological burden”, “physical burden”, “social support”,”readiness to change”, “knowledge”, and “skepticism”. Answers are given on a 4‐point Likert scale. Higher values on subscales indicate higher rehabilitation motivation except for the subscale “skepticism”. Here, higher scores indicate lower rehabilitation motivation. General quality of life was measured using the 36-item SF-36 Health Survey [18]. Higher scores indicate higher quality of life. The Facial Clinimetric Evaluation (FaCE) scale was used to measure the facial palsy-related quality of life [19, 20]. The FaCE has six independent domains: social function, facial movement, facial comfort, oral function, eye comfort, lacrimal control, and a total core incorporating all domains. Using a specific formula, a score from 0 (worst) to 100 (best) is calculated. The 24-item Liebowitz Social Anxiety Scale (LSAS), uses two subscales that address social interaction (11 items) and performance (13 items), measuring an individual's fear and avoidance of social situations over the past week. Answers are given on a 4‐point Likert scale [21]. The German version of patients’ health questionnaire (PHQ-D) was used to record depression symptoms [22]. With nine items, the DSM-IV criteria for depression are asked on a 4-point Likert scale. Higher LSAS or PHQ-D scores indicate higher social anxiety or higher depression levels, respectively. Finally, the survey included the questionnaire for technical commitment [23]. The questionnaire consists of 12 items using a 5-point Likert scale to record in subitems “technology acceptance”, “technology competence”, “technology control beliefs”, and “technology willingness”. Finally, the Technology Affinity questionnaire—attitude to and handling of electronic devices (TA-EG), was used to understand the patient’s interest, experience and trust in technology [24]. The instrument comprised 19 items covering 4 subscales and uses a 5-point Likert scale: “enthusiasm for technology”, “competence in dealing with technology”, its “positive consequences”, and “negative consequences”. Higher technical commitment subscore and higher TA-EG subscores, respectively, indicate a higher agreement.

### Facial grading

Additionally to self-ratings of facial palsy-related quality of life (with FaCE), facial motor function grading was performed using the Stennert index and the Sunnybrook Facial Grading Scale [25, 26]. The Stennert index was used because of its popularity in Germany. It was also used to classify the initial facial function at first presentation in the hospital. The observer judges facial symmetry at rest in four regional categories (0 = normal resting tone/symmetry up to 4 = no resting tone/gross asymmetry) and the motility of the facial muscles in six regional categories (0 = normal motility up to 6 = complete paralysis). The total score of the Stennert index summarizes both subscores. The Sunnybrook Facial Grading Scale was used because of its international recognition and because it allowed a separate classification of the degree of synkinesis. The Sunnybrook Facial Grading Scale is a regional weighted system that rates three subscores: resting symmetry, the degree of voluntary facial muscle movement, and involuntary muscle contraction (synkinesis). The three subscores are used to calculate a composite score (0 = total paralysis; 100 = normal function).

### Statistics

All outcome variables were analyzed with IBM SPSS statistics software (Version 25; IBM. New York) for medical statistics. Data are presented as frequencies or mean ± standard deviation (SD) if not otherwise indicated. To investigate selection bias we compared the data of patients who answered the questionnaire with the patients not responding. Pearson’s chi-square test was used for nominal data and the Mann–Whitney *U* test for metric data. McNemar test was used to compare binominal data of facial therapy types received in the past to future interests. Predictors for future interest in computer-based home therapy were explored using chi-square test for nominal data and Mann–Whitney *U* test for metric data. Predictors for the rehabilitation motivation were explored using Spearman’s correlation of PAREMO-20 subscales and all other parameters, e.g. social anxiety score. Comparisons of subgroups were only performed if a subgroup contained ≥ 10 patients. Linear regression analyses including parameters from univariate analysis and *p* < 0.05 were performed to evaluate associations related to the PAREMO-20 subscales. The significance level was set at *p* < 0.05.

## Results

### Investigation of a selection bias: comparison of study participants with the patients not responding to the survey

Sixty-nine patients with PFS answered the survey. 135 patients did not answer. The comparison of both groups is summarized in Supplement Table 1. The study participants performed more frequently facial exercises at home (*p* < 0.0001), had more therapy with a therapist (*p* < 0.0001), and received more frequently botulinum toxin injections (*p* = 0.003). Gender distribution, age, and initial severity of the palsy were not different between both groups (all *p* > 0.05). Related to the Stennert index in motion and total index, improvement of the palsy from onset to time of the survey was better for non-participants (*p* = 0.008; *p* = 0.023, respectively). The composite score of the Sunnybrook grading revealed a lower score (worse function) for study participants (*p* = 0.022).

### Baseline characteristics, socioeconomic data, motor and non-motor deficits, and interest in further therapy types of the study participants

Median age of the participants was 54 years. Most patients were females (72.5%). Median onset of facial palsy was 2.6 years ago. More details are shown in Table 1. Nearly all patients had performed mirror training at home (94.2%) or reported a prior specific facial training with a therapist (82.6%). All patients had a therapy form in direct contact with a therapist. The median number of different types of facial therapy (eye protection excluded) was 4 (range: 1–7). Other frequent therapies in the chronic phase of the disease were as follows: continued eye protection (85.5%), acupuncture (49.3%), electrostimulation (44.0%), and physical therapy with cold, heat or light (40.6%). The three most often mentioned still disturbing symptoms were as follows: Impaired eye closure (66.7%), asymmetric face (63.8%), and impaired smiling (52.2%). Stennert index and Sunnybrook grading confirmed the still affected facial motor function: Median total Stennert index and composite score of the Sunnybrook index were 4 and 41, respectively. This corresponded to patient’s self-reported facial function (Table 2): The FaCE Facial movement subscore was the lowest with a median value of 33.0, followed by the FaCE Facial comfort subscore (median: 50.0), and the FaCE Eye comfort subscore (median: 62.5). From the SF-36 subscores, SF-36 Vitality (median: 55.0), and SF-36 General health (median: 57.0) showed the worst results. LSAS and PHQ-9 showed a significant impairment in the study group. Technical commitment and technology affinity to electronic devices was moderate.

**Table 1:** Patients’ characteristics and socioeconomic data (*N* = 69)

Parameter	Absolute	%
Gender		
Female	50	72.5
Male	19	27.5
Permanent relationship		
No	14	20.3
Yes	55	79.7
Profession		
Employed	41	59.4
Retired	19	27.5
Unemployed	7	10.1
In apprentice/studying	2	2.9
Highest education		
Secondary school	26	37.7
High school	12	17.4
University	29	42.0
Other	2	2.9
Previous therapy in chronic phase of the disease		
Home mirror training	65	94.2
Supportive eye protection*	59	85.5
Facial training with therapist	57	82.6
Acupuncture	34	49.3
Electrostimulation	32	46.2
Biofeedback training	31	44.0
Light/heat/cold therapy	28	40.6
Facial training with computer	20	29.0
Eye lid surgery	14	20.3
Botulinum toxin	18	26.1
Angle of mouth surgery	2	2.9
Still most disturbing symptoms		
Impaired/uncontrolled eye closure	46	66.7
Asymmetric face	44	63.8
Impaired smiling	36	52.2
Decreased tearing	17	24.6
Increased tearing	8	11.6
	Mean ± SD	Median, range
Age, years	50.4 ± 14.2	54, 20–76
Interval onset to survey, months	5.9 ± 9.1	2.6, 0.5–60.6
Stennert index, at rest	1.1 ± 1.3	1, 0–4
Stennert index, in motion	3.3 ± 1.6	3, 1–6
Stennert index, total	4.4 ± 2.8	4, 1–10
Sunnybrook, composite	44.8 ± 20.5	41, 4–97

*SD*  Standard deviation

*Eye drops, ointment, watch glass

**Table 2:** Results of the questionnaires (*N* = 69)

	Mean ± SD	Median, range	
Facial Clinimetric Evaluation Scale (FaCE)			
FaCE Facial movement	38.1 ± 21.6	33.3, 0–91.7	
FaCE Facial comfort	50.5 ± 26.3	50.0,0–100	
FaCE Oral function	76.1 ± 25.3	87.5, 0–100	
FaCE Eye comfort	57.7 ± 31.9	62.5, 0–100	
FaCE Lacrimal control	68.8 ± 29.5	75.0, 0–100	
FaCE Social function	64.7 ± 27.5	68.6, 0–100	
FaCE Total score	57.1 ± 16.5	60.0, 13.3–92.9	
36-Item Short Form Survey (SF-36)			
SF-36 Physical functioning	83.4 ± 22.8	90.0, 5–100	
SF-36 Role physical	64.7 ± 39.4	75.0, 0–100	
SF-36 Bodily pain	70.5 ± 27.1	74.0, 0–100	
SF-36 General health	58.0 ± 23.9	57.0, 10–100	
SF-36 Vitality	54.1 ± 20.3	55.0, 10–100	
SF-36 Social role functioning	69.9 ± 26.6	75.0, 0–100	
SF-36 Emotional role functioning	66.5 ± 42.7	100.0, 0–100	
SF-36 Mental health	66.8 ± 19.5	68.0, 16–96	
SF-36 Physical health sum score	47.4 ± 9.6	48.9, 23.3–58.7	
SF-36 Mental health sum score	44.6 ± 12.6	46.9, 18.7–66.8	
Liebowitz Social Anxiety Scale (LSAS)			
LSAS Anxiety	43.4 ± 15.8	40.8, 24–96	
LSAS Avoidance	44.7 ± 14.9	41.7, 24–96	
LSAS Total	87.8 ± 30.1	82.0, 48–192	
Patient Health Questionnaire (PHQ)-9	5.8 ± 4.9	4, 0–20	
Technical commitment			
Technology acceptance	2.9 ± 0.5	2.8, 1.8–4.0	
Technology competence	3.0 ± 0.6	3.0, 1.3–4.0	
Technology control beliefs	2.8 ± 0.7	2.8, 1.0–4.8	
Technology willingness	2.9 ± 0.5	3.0, 1.4–3.7	
Technology affinity to electronic devices			
Enthusiasm	3.0 ± 0.9	3.0, 1.0–5.0	
Subjective competency	3.4 ± 0.8	3.3, 1.0–5.0	
Negative impacts	2.6 ± 0.5	2.6, 1.6–3.8	
Positive impacts	3.1 ± 0.6	3.1, 2.0–4.5	
PAREMO-20			
Psychological burden	6.1 ± 2.6	5, 3–12	
Physical burden	9.6 ± 3.9	10, 4–16	
Social support	8.6 ± 3.5	8, 4–16	
Readiness to change	5.8 ± 2.7	6, 3–12	
Knowledge	9.0 ± 2.7	9, 3–12	
Skepticism	7.1 ± 2.5	7.5, 3–12	

*PAREMO* Questionnaire for patient rehabilitation motivation

**Fig. 1:**
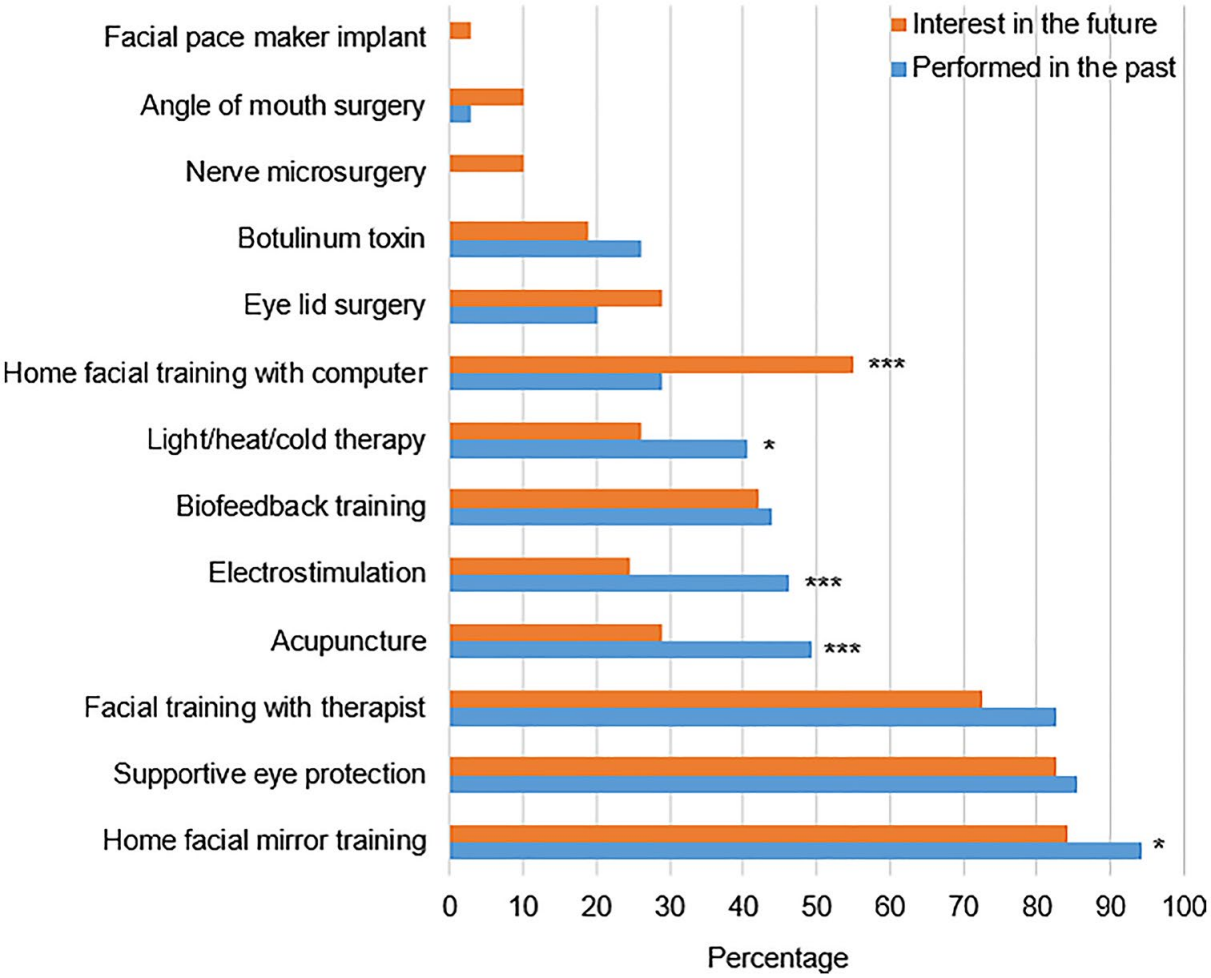
Comparison between future interests in different facial therapy types (red) to performed therapy in the past (blue). Calculations via McNemar test: * = *p* < 0.05; *** = *p* < 0.001

All patients showed interest in further therapy (Supplement Table 2), mostly in the form of home mirror therapy (84.1%), continual eye protection (82.6%), facial training with therapist (72.5%), with a computer (55.1%), and biofeedback therapy (42.0%). Compared to the past, the future interest in home facial mirror training, acupuncture, electrostimulation, and any light/heat/cold therapy decreased significantly (all *p* < 0.05; Fig. 1), while the interest in computer-assisted home facial training increased significantly (p < 0.0001). Therefore, factors with association with this wish were further explored (Supplement Table 3). Patients with future interest in computer-assisted facial therapy showed a lower FaCE Eye comfort subscore (*p* = 0.044), lower SF-36 Vitality subscore (*p* = 0.008), lower SF-36 Social functioning (*p* = 0.009), lower SF-36 Role functioning (*p* = 0.010), lower SF-36 Mental health (*p* = 0.005, leading also to a lower SF-36 Mental summary score (*p* = 0.002).

### Rehabilitation motivation domains and influencing factors

Table 2 lists the results of the PAREMO-20 subscores. The two highest scores were the Physical burden subscore (median: 10) and the Knowledge subscore (median: 9). The PAREMO-20 subscores Psychological burden, Physical burden, and Readiness to change showed the highest bivariate correlation to each other (Supplement Table 4). The correlations between the six PAREMO-20 subscores and patients’ characteristics and the other assessments did not show a uniform picture (Supplement Table 5).

Most relevant correlations (*r* > 0.5) were seen between low FaCE Social function, or low FaCE Total score versus high Psychological burden or Physical burden subscore. Several SF-36 subscores and the LSAS subscores were negatively correlated with Psychological burden or Physical burden subscore. The SF-36 General health or SF-36 Vitality domain also correlated with Readiness to change subscore. PHQ-9 was negatively correlated to Psychological burden, Physical burden or Readiness to change subscore. Technology commitment, technology affinity, facial grading, or the therapy interests of the patients did not show any high correlation.

The results of the multivariate linear regression analyses for independent associations to the PAREMO-20 subscores are shown in Table 3. For the Psychological burden subscore, SF36 Emotional role was the highest negative correlative factor (beta = − 0.026; 95% confidence interval [95% CI] = − 0.040 to − 0.012; *p* < 0.0001). For the Physical burden subscore, SF-36 General health was the highest negative correlative factor (beta = − 0.048; 95% CI = − 0.088 to − 0.009; *p* = 0.018). Working (beta = 2.149; 95% CI = 0.184 to 4.114; *p* = 0.033) and a steady relationship (beta = 1.823; 95% CI = 0.192 to 3.453; *p* = 0.029) predicted the Social Support Subscore. A predictor for readiness for change was not found. Younger age was associated with a higher Knowledge subscore (beta = − 0.070; 95% CI = − 0.125 to − 0.015; *p* = 0.013). Higher positive impacts of technology affinity was correlated to lower Knowledge subscore (beta = − 1.700; 95% CI = − 3.082 to − 0.317; *p* = 0.017). Finally, a lower SF-36 Role physical subscore (beta = − 0.023; 95% CI = − 0.046 to − 0.001; *p* = 0.045) and a lower SF-36 General health (beta = − 0.038, 95% CI = − 0.067 to − 0.008; *p* = 0.013) were correlated to a higher Skepticism subscore.

**Table 3:** Multivariate linear regression analysis for independent associations with the PAREMO-20 subscores

Measure	Beta	95% CI lower	95% CI upper	Stand.* beta	p**
PAREMO-20 Psychological burden					
PROMs; *R*^*2*^ = 0.850; *p* < 0.0001					
FaCE Facial comfort	− 0.007	− 0.023	0.009	− 0.068	0.410
FaCE Eye comfort	− 0.014	− 0.028	− 0.001	− 0.177	**0.042**
FaCE Social function	− 0.013	− 0.037	0.011	− 0.135	0.294
SF-36 Role physical	0.015	0.001	0.029	0.229	**0.041**
SF-36 Bodily pain	− 2E-05	− 0.021	0.021	0.000	0.998
SF-36 General health	− 0.018	− 0.039	0.002	− 0.170	0.080
SF-36 Vitality	0.043	0.004	0.082	0.343	0.032
SF-36 Social role functioning	− 0.011	− 0.041	0.019	− 0.116	0.461
SF-36 Emotional role functioning	− 0.026	− 0.040	− 0.012	− 0.438	** < 0.0001**
SF-36 Mental health	− 0.041	− 0.092	0.009	− 0.313	0.108
LSAS Anxiety	0.017	− 0.048	0.083	0.105	0.602
LSAS Avoidance	− 0.029	− 0.102	0.045	− 0.166	0.435
PHQ-9	0.133	− 0.026	0.293	0.250	0.100
PAREMO-20 Physical burden					
Socioeconomic aspects; *R*^*2*^ = 0.326; *p* = 0.025					
Age, years	0.062	− 0.003	0.126	0.227	0.062
Relationship (0 = no; 1 = yes)	1.770	− 0.503	4.043	0.186	0.125
PROMs; *R*^*2*^ = 0.768; *p* < 0.0001					
FaCE Facial comfort	− 0.030	− 0.063	0.003	− 0.204	0.073
FaCE Oral function	0.022	− 0.066	0.110	0.144	0.616
FaCE Eye comfort	− 0.029	− 0.055	− 0.003	− 0.246	**0.027**
FaCE Lacrimal control	− 0.017	− 0.094	0.061	− 0.118	0.669
FaCE Social function	− 0.024	− 0.078	0.029	− 0.175	0.365
SF-36 Physical functioning	− 0.013	− 0.063	0.036	− 0.079	0.594
SF-36 Role physical	0.009	− 0.022	0.039	0.087	0.577
SF-36 Bodily pain	0.004	− 0.037	0.046	0.029	0.842
SF-36 General health	− 0.048	− 0.088	− 0.009	− 0.300	**0.018**
SF-36 Vitality	− 0.003	− 0.078	0.072	− 0.015	0.940
SF-36 Social role functioning	− 0.045	− 0.111	0.020	− 0.314	0.173
SF-36 Emotional role functioning	0.000	− 0.028	0.027	− 0.002	0.990
SF-36 Mental health	0.010	− 0.086	0.105	0.049	0.840
LSAS Anxiety	− 0.039	− 0.164	0.086	− 0.160	0.534
LSAS Avoidance	0.028	− 0.112	0.168	0.110	0.686
PHQ-9	0.020	− 0.283	0.323	0.025	0.896
PAREMO-20 Social support					
Socioeconomic aspects; *R*^*2*^ = 0.438; *p* = 0.003					
Gender (0 = male; 1 = female)	− 1.724	− 3.468	0.020	− 0.220	0.053
Relationship (0 = no; 1 = yes)	2.149	0.184	4.114	0.247	**0.033**
Working (0 = no; 1 = yes)	− 1.823	− 3.453	− 0.192	− 0.252	**0.029**
PROMs; *R*^*2*^ = 0.569; *p* = 0.028					
FaCE Eye comfort	− 0.008	− 0.037	0.021	− 0.073	0.578
FaCE Social function	− 0.017	− 0.070	0.036	− 0.128	0.531
SF-36 Physical functioning	− 0.038	− 0.090	0.014	− 0.243	0.147
SF-36 Role physical	− 0.007	− 0.041	0.028	− 0.073	0.703
SF-36 Bodily pain	− 0.009	− 0.054	0.035	− 0.072	0.669
SF-36 General health	− 0.042	− 0.086	0.003	− 0.277	0.065
SF-36 Vitality	− 0.012	− 0.097	0.073	− 0.069	0.779
SF-36 Social role functioning	0.016	− 0.053	0.085	0.120	0.646
SF-36 Mental health	0.056	− 0.047	0.159	0.306	0.281
LSAS Anxiety	− 0.004	− 0.144	0.136	− 0.017	0.957
LSAS Avoidance	0.048	− 0.110	0.206	0.198	0.548
PHQ-9	0.058	− 0.282	0.398	0.079	0.733
Facial grading					
Stennert index, at rest, initial	− 0.455	− 2.438	1.529	− 0.178	0.648
Stennert index, in motion, initial	1.071	− 0.476	2.617	0.523	0.171
Stennert index, at rest	1.102	− 0.797	3.002	0.406	0.250
Stennert index, in motion	− 0.751	− 2.258	0.755	− 0.336	0.322
Sunnybrook, composite	− 0.001	− 0.076	0.075	− 0.004	0.986
PAREMO-20 Readiness to change					
PROMs; *R*^*2*^ = 0.699; *p* < 0.0001					
FaCE Social function	0.002	− 0.032	0.036	0.022	0.900
SF-36 Physical functioning	− 0.009	− 0.044	0.026	− 0.079	0.598
SF-36 Role physical	− 0.002	− 0.024	0.020	− 0.035	0.833
SF-36 Bodily pain	0.002	− 0.027	0.030	0.017	0.909
SF-36 General health	− 0.027	− 0.055	0.002	− 0.239	0.067
SF-36 Vitality	0.010	− 0.045	0.065	0.074	0.727
SF-36 Social role functioning	− 0.006	− 0.052	0.040	− 0.060	0.794
SF-36 Emotional role functioning	− 0.017	− 0.038	0.003	− 0.279	0.093
SF-36 Mental health	− 0.038	− 0.108	0.031	− 0.281	0.276
LSAS Anxiety	− 0.027	− 0.118	0.064	− 0.160	0.556
LSAS Avoidance	0.025	− 0.076	0.127	0.142	0.618
PHQ-9	− 0.004	− 0.223	0.216	− 0.007	0.972
Technology affinity; *R*^*2*^ = 0.199; *p* = 0.267					
TA Subjective competency	− 0.708	− 1.584	0.167	− 0.209	0.111
TA Positive impacts	0.168	− 1.016	1.353	0.037	0.777
PAREMO-20 Knowledge					
Socioeconomic aspects; *R*^*2*^ = 0.406; *p* = 0.003					
Age, years	− 0.070	− 0.125	− 0.015	− 0.363	**0.013**
Working (0 = no; 1 = yes)	0.360	− 1.235	1.956	0.064	0.653
Prior therapy chronic phase; *R*^*2*^ = 0.460; *p* = 0.002					
Facial training therapist	2.441	0.683	4.200	0.328	**0.007**
Biofeedback training	1.343	− 0.219	2.904	0.241	0.091
Facial training computer	0.036	− 1.384	1.456	0.007	0.960
PROMs; *R*^*2*^ = 0.356; *p* = 0.012					
FaCE Oral function	− 0.033	− 0.097	0.031	− 0.301	0.313
FaCE Lacrimal control	0.057	0.002	0.112	0.614	**0.042**
Technology affinity; *R*^*2*^ = 0.407; *p* = 0.009					
TA Subjective competency	0.499	− 0.387	1.386	0.139	0.265
TA Negative impacts	0.697	− 0.483	1.876	0.144	0.242
TA Positive impacts	− 1.700	− 3.082	− 0.317	− 0.288	**0.017**
Therapy interests; *R*^*2*^ = 0.512; *p* = 0.002					
Facial training with therapist	0.845	− 0.752	2.441	0.133	0.294
Facial training with computer	1.743	− 0.131	3.616	0.332	0.068
Biofeedback training	0.605	− 1.329	2.539	0.115	0.533
Botulinum toxin	0.356	− 1.291	2.004	0.056	0.666
PAREMO-20 Skepticism					
PROMs; *R*^*2*^ = 0.613; *p* = 0.006					
FaCE Facial movement	− 0.018	− 0.045	0.009	− 0.155	0.192
FaCE Facial comfort	− 0.021	− 0.044	0.001	− 0.226	0.057
FaCE Social function	− 0.007	− 0.043	0.029	− 0.074	0.711
SF-36 Physical functioning	− 0.003	− 0.037	0.030	− 0.029	0.849
SF-36 Role physical	− 0.023	− 0.046	− 0.001	− 0.364	**0.045**
SF-36 General health	− 0.038	− 0.067	− 0.008	− 0.359	**0.013**
SF-36 Vitality	0.003	− 0.053	0.060	0.027	0.907
SF-36 Social role functioning	0.033	− 0.014	0.081	0.357	0.165
SF-36 Mental health	0.035	− 0.033	0.103	0.265	0.311
LSAS Anxiety	0.056	− 0.039	0.150	0.353	0.243
LSAS Avoidance	− 0.062	− 0.169	0.045	− 0.372	0.249
PHQ-9	0.181	− 0.053	0.414	0.339	0.127

*PROM* Patient-reported outcome measures,* FaCE* Facial Clinimetric Evaluation, *SF* Short Form, *LSAS* Liebowitz Social Anxiety Scale, *PHQ* Patient Health Questionnaire, *TA* Technology affinity

*Standardized beta

**p-values < 0.05 in bold

## Discussion

The motivation of further rehabilitation of patients with PFS is an under-researched topic. The presented cohort still showed after a median time of 2.6 years after onset of the palsy a relevant facial motor and non-motor dysfunction. The reduced quality of life values (SF-36, FaCE) are within the range of prior studies. The reported values for LSAS and PHQ-9 are much higher in patients with PFS than in the normal population [19, 27–30]. The study group represents a selection of patients referred to a specialist center. All participants already had in the chronic phase of the disease at least one of internationally accepted types of non-surgical facial therapy [11, 31]. Additionally, nearly all patients performed a supplementary facial training at home.

In general, the access to a specialized therapist is limited [11]. The efficacy of any facial training is related to the duration of each session and frequency [31]. Therefore, facial therapy for patients with PFS is normally combined with home training [31]. Patient’s adherence to such a home training was not sufficiently investigated so far, but patients’ barriers are known: fitting exercises into daily life, use of a mirror, and lack of regular feedback by a therapist [11]. These might be reasons why the motivation to perform a classical home training in the future was much lower in the present study in comparison to the frequent use in the past. Instead, there was a significant the interest in home computer-based facial training. The discussion about such training forms including telerehabilitation is gaining an entirely new significance by the COVID-19 pandemic [32]. A previous analysis had already shown that the patients with facial palsy would find it very attractive to perform in the future facial therapy with an emotion-sensitive training system. Like in the present study, patients with more severe impairment of facial expression and psychosocial impairment rated significantly higher acceptance with such innovative systems [12].

The focus of this paper was to investigate the different dimensions of the patients’ rehabilitation motivation. Using the PAREMO-20, the motivation dimensions Physical Burden, Social Support, and Knowledge were still high in our study collective even after long duration of PFS and much experience with standard facial therapy types. PAREMO data for the acute phase of facial palsy or data for any other directly comparable disease do not exist so far. The values in the Physical Burden, Social Support, and Knowledge dimensions were nearly as high as reported directly after herniated disc surgery or for cardiological patients after surgery [6, 33]. The Skepticism dimension was higher than reported for patients after acute surgery [33]. Probably, this reflects that our patients underwent already several rehabilitation therapies with insufficient success, at least less success than subjectively expected. The expressed interest in continual rehabilitation, especially in patients with prior experience with facial therapy with a therapist, higher physical burden, especially dysfunctional eye closure, make clear that new and additional therapy concepts have to be developed for a continual facial therapy. A possible solution could be the development and validation of home-based sensor-based digital technology at best in combination with a remote monitoring function for the involved therapist [11, 12, 34]. The subgroup of the patients with high psychosocial burden would probably profit from an integration of remotely communicated, therapist-delivered psychotherapy [35].

Although a good characterized sample was evaluated, the study has the typical limitations of a retrospective analysis. A selection bias beyond the per se selection of motivated patients seems to be negligible but cannot be ruled out. So far treatment of patients with PFS, whether classically face-to-face with a therapist or with any innovative home-based approaches, is mainly focused on facial motor disturbances. Next steps should address better integration of direct treatment of facial non-motoric disturbances into the treatment concepts for patients with PFS.

## Conclusions

Postparalytic facial synkinesis (PFS) leads to persistent worse general and disease-specific quality of life as well as a continual interest in rehabilitation therapy beyond the second year after onset of the acute facial palsy. The patients show a high interest in innovative digital solutions for facial rehabilitation. The non-motoric facial dysfunctions enforce the rehabilitation motivation. Boosted by the pandemic, the future focus will be in home-based sensor-based digital technology solutions with remote monitoring by the facial therapist allowing frequent intensive training sessions. A combination with remote psychotherapy could be useful to threat also the high levels so social anxiety and depressions.

## Supplementary Information

Below is the link to the electronic supplementary material.

Supplementary file1 (DOCX 95 kb)
