# Supplementary material for: Continual rehabilitation motivation of patients with postparalytic facial nerve syndrome
Source: Eur Arch Otorhinolaryngol. 2021 May 24;279(1):481–91. doi: 10.1007/s00405-021-06895-2 (PMC8141409; doi:10.1007/s00405-021-06895-2)
Supplement: Supplementary file 1 — Supplementary file1 (DOCX 95 kb) [file 405_2021_6895_MOESM1_ESM.docx]

**Supplement Tables**

**Supplement Table 1**

| **Supplement Table 1.** Comparison of (n=204) study participants (N=69) and patients not responding to the survey (N=135). | | | | |
| --- | --- | --- | --- | --- |
|  | **Study participants** | **Non-responders** | **p*** |  |
| **Parameter** | **Absolute (N=69)** | **Absolute (N=135)** |  |  |
| Gender |  |  | 0.145 |  |
| Female | 50 | 85 |  |  |
| Male | 19 | 51 |  |  |
| **Therapy during acute phase** |  |  |  |  |
| Drug therapy |  |  | 0.754 |  |
| No | 22 | 46 |  |  |
| Yes | 47 | 89 |  |  |
| Cornea protection |  |  | 0.171 |  |
| No | 51 | 87 |  |  |
| Yes | 18 | 48 |  |  |
| **Therapy in chronic phase** |  |  |  |  |
| Facial exercises at home |  |  | **<0.0001** |  |
| No | 2 | 51 |  |  |
| Yes | 67 | 84 |  |  |
| Any therapy with therapist |  |  | **<0.0001** |  |
| No | 5 | 64 |  |  |
| Yes | 64 | 71 |  |  |
| Botulinumtoxin injection |  |  | **0.003** |  |
| No | 50 | 120 |  |  |
| Yes | 19 | 15 |  |  |
|  | **Mean±SD** | **Mean±SD** |  |  |
| Age, years | 50.4±14.2 | 47.7±16.3 | 0.080 |  |
| Interval onset to survey, months | 5.9±9.1 | 5.1±8.7 | 0.642 |  |
| **Initial severity of facial palsy** |  |  |  |  |
| Stennert index, at rest | 1.4±1.4 | 1.4±1.4 | 0.650 |  |
| Stennert index, in motion | 3.7±1.7 | 3.6±1.8 | 0.501 |  |
| Stennert index, total | 5.1±3.0 | 4.9±3.0 | 0.091 |  |
| **Severity of facial palsy at survey** |  |  |  |  |
| Stennert index, at rest | 1.1±1.3 | 0.9±1.3 | 0.208 |  |
| Stennert index, in motion | 3.3±1.6 | 2.8±1.6 | 0.073 |  |
| Stennert index, total | 4.4±2.8 | 3.6±2.8 | 0.106 |  |
| Sunnybrook, composite | 44.8±20.5 | 53.6±26.1 | **0.022** |  |
| **Change** severity onset to survey** |  |  |  |  |
| ΔStennert index, at rest | 0.3±0.8 | 0.4±1.0 | 0.222 |  |
| ΔStennert index, in motion | 0.4±1.1 | 0.7±1.5 | **0.008** |  |
| ΔStennert index, total | 0.8±1.7 | 1.1±2.4 | **0.023** |  |

SD=standard deviation; *p-values <0.05 in bold; **positive value = improvement

**Supplement Table 2**

| **Supplement Table 2.** Interest in further therapy (N=69). | | |
| --- | --- | --- |
| **Interest in further therapy types** | **Absolute** | **%** |
| Home facial mirror training | 58 | 84.1 |
| Supportive eye therapy | 57 | 82.6 |
| Facial training with therapist | 50 | 72.5 |
| Home facial training with computer | 38 | 55.1 |
| Biofeedback training | 29 | 42.0 |
| Eye lid surgery | 20 | 29.0 |
| Acupuncture | 18 | 29.0 |
| Light/heat/cold therapy | 18 | 26.1 |
| Electrostimulation | 17 | 24.6 |
| Botulinum toxin | 13 | 18.8 |
| Nerve surgery | 7 | 10.1 |
| Angle of mouth surgery | 7 | 10.1 |
| Facial pace maker surgery | 2 | 2.9 |

*eye drops, ointment, watch glass

**Supplement Table 3**

| **Supplement Table 3.** Comparison of patients with future interest in computer-based home facial therapy and patients without this interest (N=69). | | | |
| --- | --- | --- | --- |
| **Parameter** | **Interest in computer-Based therapy**  **(N=38)** | **No interest in computer-based therapy**  **(N=31)** | **p*** |
| Gender |  |  | 0.182 |
| Female | 30 | 20 |  |
| Male | 8 | 11 |  |
| Relationship |  |  | 0.303 |
| No | 6 | 8 |  |
| Yes | 32 | 23 |  |
| University degree |  |  | 0.320 |
| No | 20 | 20 |  |
| Yes | 18 | 11 |  |
| Working |  |  | 0.734 |
| No | 15 | 11 |  |
| Yes | 23 | 20 |  |
|  | **Mean±SD** | **Mean±SD** |  |
| Age, years | 48.5±13.7 | 52.7±14.7 | 0.163 |
| Interval to onset, months | 6.0±7.1 | 5.5±10.9 | 0.066 |
| **PROMs** |  |  |  |
| FaCE Facial movement | 37.4±20.5 | 38.9±23.1 | 0.901 |
| FaCE Facial comfort | 50.2±25.2 | 50.8±27.9 | 0.752 |
| FaCE Oral function | 77.6±28.1 | 74.2±21.6 | 0.129 |
| FaCE Eye comfort | 51.0±31.6 | 66.3±30.7 | **0.044** |
| FaCE Lacrimal control | 73.0±29.3 | 63.7±29.5 | 0.134 |
| FaCE Social function | 61.6±30.0 | 68.6±24.0 | 0.348 |
| FaCE Total score | 55.5±18.6 | 59.2±13.6 | 0.686 |
| SF-36 Physical functioning | 79.5±27.1 | 88.2±15.1 | 0.234 |
| SF-36 Role physical | 55.7±40.8 | 75.8±35.1 | 0.055 |
| SF-36 Bodily pain | 66.5±30.3 | 75.4±22.0 | 0.336 |
| SF-36 General health | 55.7±24.2 | 60.8±23.5 | 0.308 |
| SF-36 Vitality | 48.0±21.4 | 61.5±16.3 | **0.008** |
| SF-36 Social functioning | 62.2±28.4 | 79.5±21.1 | **0.009** |
| SF-36 Role emotional | 53.5±46.8 | 82.5±31.1 | **0.010** |
| SF-36 Mental health | 60.6±21.1 | 74.4±14.9 | **0.005** |
| SF-36 Physical summary score | 46.4±10.5 | 48.7±8.4 | 0.455 |
| SF-36 Mental summary score | 40.4±13.3 | 49.7±9.5 | **0.002** |
| LSAS Anxiety | 46.2±16.7 | 39.9±14.1 | 0.090 |
| LSAS Avoidance | 47.0±16.4 | 41.9±12.6 | 0.201 |
| LSAS social anxiety Total | 93.1±32.6 | 81.0±25.6 | 0.106 |
| PHQ-9 depressive symptoms | 6.9±5.5 | 4.4±3.7 | 0.070 |
| **Technology commitment** |  |  |  |
| Technology acceptance | 3.0±0.5 | 2.8±0.6 | 0.232 |
| Technology competence | 3.0±0.5 | 2.9±0.6 | 0.342 |
| Technology control beliefs | 2.8±0.7 | 2.8±0.8 | 0.879 |
| Technology willingness | 2.9±0.4 | 2.8±0.5 | 0.514 |
| **Technology affinity** |  |  |  |
| TA Enthusiasm | 3.1±0.9 | 2.9±1.0 | 0.586 |
| TA Subjective competency | 3.4±0.7 | 3.3±0.8 | 0.511 |
| TA Negative impacts | 3.2±0.6 | 2.6±0.5 | 0.304 |
| TA Positive impacts | 2.5±0.4 | 3.1±0.6 | 0.273 |
| **Facial grading** |  |  |  |
| Stennert index, at rest, initial | 1.3±1.3 | 1.7±1.4 | 0.232 |
| Stennert index, in motion, initial | 3.5±1.6 | 3.8±1.9 | 0.404 |
| Stennert index, total, initial | 4.8±2.8 | 5.5±3.1 | 0.375 |
| Stennert index, at rest | 1.0±1.3 | 1.3±1.4 | 0.388 |
| Stennert index, in motion | 3.2±1.4 | 3.4±1.8 | 0.615 |
| Stennert index, total | 4.1±2.6 | 4.6±3.0 | 0.592 |
| Sunnybrook, composite | 46.8±19.3 | 42.2±21.9 | 0.262 |

SD=standard deviation; *p-values <0.05 in bold; PROM = Patient-reported outcome measures; FaCE = Facial Clinimetric Evaluation; SF = Short Form; LSAS = Liebowitz Social Anxiety Scale; PHQ = Patient Health Questionnaire; TA = Technology affinity

**Supplement Table 4**

| **Supplement Table 4.** Bivariate correlation between patients’ characteristics and assessments versus PAREMO-20 subscores.* | | | | | |
| --- | --- | --- | --- | --- | --- |
| **Parameter** | **Physical burden**  **r p** | **Social support**  **r p** | **Readiness to change**  **r p** | **Knowledge**  **r p** | **Skepticism**  **r p** |
| Psychological burden | 0.601 **<0.0001** | 0.217 0.073 | 0.638 **<0.0001** | -0.158 0.195 | 0.171 0.161 |
| Physical burden |  | 0.544 **<0.0001** | 0.630 **<0.0001** | -0.145 0.234 | 0.384 **0.001** |
| Social support |  |  | 0.419 **<0.0001** | -0.137 0.262 | 0.235 0.052 |
| Readiness to change |  |  |  | -0.240 **0.047** | 0.288 **0.016** |
| Knowledge |  |  |  |  | -0.479 **<0.0001** |
| Skepticism |  |  |  |  |  |

NA = not applicable. *p-values <0.05 in bold

**Supplement Table 5**

| **Supplement Table 5.** Bivariate correlation analyses between patients’ characteristics and assessments versus PAREMO-20 subscores.* | | | | | | |
| --- | --- | --- | --- | --- | --- | --- |
| **Parameter** | **Psychological burden**  **r p** | **Physical burden**  **r p** | **Social support**  **r p** | **Readiness to change**  **r p** | **Knowledge**  **r p** | **Skepticism**  **r p** |
| **Socioeconomic aspects** |  |  |  |  |  |  |
| Gender (0=male; 1=female) | -0.057 0.643 | -0.104 0.396 | -0.252 **0.037** | -0.139 0.253 | 0.158 0.194 | -0.140 0.251 |
| Age, years | 0.030 0.806 | 0.247 **0.041** | 0.237 0.050 | 0.119 0.330 | -0.374 **0.002** | 0.223 0.065 |
| Interval to onset, months | -0.116 0.344 | -0.122 0.317 | -0.077 0.531 | -0.141 0.247 | 0.065 0.598 | -0.174 0.154 |
| Relationship (0=no; 1=yes) | 0.069 0.576 | 0.238 **0.049** | 0.282 **0.019** | 0.135 0.270 | -0.014 0.910 | 0.303 **0.011** |
| University degree (0=no; 1=yes) | -0.097 0.429 | -0.107 0.384 | 0.112 0.358 | -0.120 0.326 | 0.125 0.305 | 0.009 0.941 |
| Working (0=no; 1=yes) | -0.124 0.309 | -0.184 0.131 | -0.267 **0.027** | -0.204 0.092 | 0.267 **0.027** | -0.212 0.081 |
| **Prior therapy chronic phase**** |  |  |  |  |  |  |
| Supportive eye protection | 0.043 0.736 | 0.018 0.891 | -0.027 0.832 | -0.066 0.606 | 0.011 0.930 | 0.189 0.134 |
| Facial training with therapist | -0.064 0.608 | 0.021 0.867 | -0.107 0.395 | -0.114 0.360 | 0.372 **0.002** | -0.132 0.290 |
| Acupuncture | -0.079 0.540 | 0.064 0.620 | -0.119 0.352 | -0.181 0.155 | 0.210 0.099 | 0.016 0.901 |
| Electrostimulation | -0.089 0.485 | -0.013 0.920 | 0.092 0.469 | -0.013 0.919 | 0.103 0.416 | -0.053 0.675 |
| Biofeedback training | 0.095 0.436 | 0.138 0.257 | -0.148 0.224 | 0.145 0.236 | 0.252 **0.037** | -0.111 0.364 |
| Light/heat/cold therapy | 0.075 0.551 | 0.023 0.853 | 0.049 0.696 | 0.018 0.888 | 0.169 0.179 | -0.049 0.699 |
| Facial training with computer | 0.084 0.503 | 0.254 **0.040** | 0.028 0.824 | 0.258 **0.036** | 0.333 **0.006** | -0.010 0.938 |
| Eye lid surgery | 0.080 0.538 | 0.059 0.650 | 0.148 0.254 | 0.125 0.338 | 0.034 0.796 | 0.062 0.635 |
| Botulinum toxin | 0.026 0.835 | 0.011 0.931 | -0.180 0.149 | 0.147 0.240 | 0.138 0.268 | -0.045 0.717 |
| **PROMs** |  |  |  |  |  |  |
| FaCE Facial movement | -0.160 0.191 | -0.170 0.165 | -0.242 0.047 | -0.161 0.191 | 0.144 0.242 | -0.056 **0.035** |
| FaCE Facial comfort | -0.250 **0.038** | -0.284 **0.018** | -0.013 0.916 | -0.192 0.114 | 0.237 0.050 | -0.332 **0.005** |
| FaCE Oral function | -0.208 0.087 | -0.287 **0.017** | -0.217 0.073 | -0.227 0.061 | 0.298 **0.013** | -0.234 0.053 |
| FaCE Eye comfort | -0.349 **0.004** | -0.494 **<0.0001** | -0.264 **0.029** | -0.144 0.240 | 0.031 0.800 | -0.100 0.415 |
| FaCE Lacrimal control | -0.159 0.191 | -0.257 **0.033** | -0.157 0.198 | -0.184 0.131 | 0.264 **0.028** | -0.224 0.064 |
| FaCE Social function | -0.590 **<0.0001** | -0.594 **<0.0001** | -0.292 **0.015** | -0.452 **<0.0001** | 0.060 0.621 | -0.251 **0.037** |
| FaCE Total score | -0.513 **<0.0001** | -0.603 **<0.0001** | -0.313 **0.009** | -0.350 **0.003** | 0.236 0.051 | -0.382 **0.001** |
| SF-36 Physical functioning | -0.227 0.060 | -0.439 **<0.0001** | -0.418 **<0.0001** | -0.386 **0.001** | 0.122 0.317 | -0.313 **0.009** |
| SF-36 Role physical | -0.328 **0.006** | -0.485 **<0.0001** | -0.403 **0.001** | -0.486 **<0.0001** | -0.021 0.862 | -0.389 **0.001** |
| SF-36 Bodily pain | -0.448 **<0.0001** | -0.469 **<0.0001** | -0.315 **0.008** | -0.448 **<0.0001** | 0.207 0.088 | -0.221 0.068 |
| SF-36 General health | -0.467 **<0.0001** | -0.578 **<0.0001** | -0.449 **<0.0001** | -0.562 **<0.0001** | 0.160 0.189 | -0.422 **<0.0001** |
| SF-36 Vitality | -0.518 **<0.0001** | -0.488 **<0.0001** | -0.287 **0.017** | -0.584 **<0.0001** | 0.039 0.747 | -0.264 **0.028** |
| SF-36 Social functioning | -0.620 **<0.0001** | -0.666 **<0.0001** | -0.347 **0.003** | -0.622 **<0.0001** | 0.025 0.836 | -0.222 **0.067** |
| SF-36 Role emotional | -0.720 **<0.0001** | -0.439 **<0.0001** | -0.181 0.136 | -0.605 **<0.0001** | 0.052 0.669 | -0.125 0.307 |
| SF-36 Mental health | -0.644 **<0.0001** | -0.533 **<0.0001** | -0.290 **0.016** | -0.657 **<0.0001** | 0.015 0.900 | -0.270 **0.025** |
| SF-36 Physical summary score | -0.247 **0.040** | -0.523 **<0.0001** | -0.491 **<0.0001** | -0.432 **<0.0001** | 0.126 0.303 | -0.418 **<0.0001** |
| SF-36 Mental summary score | -0.732 **<0.0001** | -0.500 **<0.0001** | -0.222 0.067 | -0.624 **<0.0001** | -0.042 0.730 | -0.156 0.199 |
| LSAS Anxiety | 0.529 **<0.0001** | 0.493 **<0.0001** | 0.328 **0.006** | 0.409  **0.001** | 0.008 0.948 | 0.260 **0.032** |
| LSAS Avoidance | 0.531 **<0.0001** | 0.552 **<0.0001** | 0.327 **0.006** | 0.490 **<0.0001** | -0.098 0.424 | 0.260 **0.031** |
| LSAS Total | 0.550 **<0.0001** | 0.543 **<0.0001** | 0.351 **0.003** | 0.466 **<0.0001** | -0.040 0.743 | 0.276 **0.023** |
| PHQ-9 | 0.587 **<0.0001** | 0.545 **<0.0001** | 0.328 **0.006** | 0.614 **<0.0001** | -0.085 0.489 | 0.322 **0.007** |
| **Technology commitment** |  |  |  |  |  |  |
| Technology acceptance | -0.019 0.875 | 0.013 0.917 | 0.007 0.954 | -0.048 0.698 | -0.002 0.987 | 0.112 0.358 |
| Technology competence | -0.091 0.457 | -0.016 0.899 | -0.015 0.902 | -0.034 0.780 | 0.079 0.518 | -0.002 0.990 |
| Technology control beliefs | 0.092 0.453 | 0.069 0.576 | 0.174 0.152 | 0.163 0.182 | -0.128 0.293 | 0.093 0.449 |
| Technology willingness | 0.042 0.732 | 0.042 0.733 | 0.078 0.526 | 0.049 0.689 | -0.053 0.666 | 0.087 0.478 |
| **Technology affinity** |  |  |  |  |  |  |
| TA Enthusiasm | -0.232 0.055 | -0.178 0.144 | -0.194 0.110 | -0.198 0.103 | 0.187 0.123 | -0.159 0.191 |
| TA Subjective competency | -0.246 **0.041** | -0.214 0.078 | -0.283 **0.019** | -0.262 **0.030** | 0.271 **0.024** | -0.182 0.135 |
| TA Negative impacts | -0.138 0.260 | -0.138 0.261 | -0.043 0.730 | -0.059 0.634 | 0.244 **0.045** | -0.067 0.586 |
| TA Positive impacts | 0.238 0.050 | 0.194 0.113 | 0.214 0.079 | 0.281 **0.020** | -0.326 **0.007** | 0.230 0.059 |
| **Facial grading** |  |  |  |  |  |  |
| Stennert index, at rest, initial | -0.063 0.612 | 0.150 0.222 | 0.383 **0.001** | -0.072 0.559 | -0.101 0.411 | 0.128 0.298 |
| Stennert index, in motion, initial | -0.069 0.574 | 0.109 0.377 | 0.377 **0.002** | -0.105 0.396 | -0.017 0.889 | -0.002 0.986 |
| Stennert index, total, initial | -0.095 0.441 | 0.111 0.367 | 0.398 **0.001** | -0.105 0.394 | -0.050 0.685 | 0.040 0.748 |
| Stennert index, at rest | -0.025 0.838 | 0.110 0.374 | 0.286 **0.019** | -0.141 0.256 | -0.047 0.703 | 0.134 0.279 |
| Stennert index, in motion | -0.039 0.750 | 0.133 0.279 | 0.263 **0.030** | -0.087 0.480 | 0.029 0.813 | -0.036 0.773 |
| Stennert index, total | -0.044 0.719 | 0.139 0.260 | 0.287 **0.018** | -0.109 0.378 | 0.007 0.955 | 0.007 0.954 |
| Sunnybrook, composite | -0.119 0.352 | -0.047 0.714 | -0.251 **0.047** | 0.019 0.881 | 0.092 0.474 | -0.094 0.466 |
| **Future interest in therapy **** |  |  |  |  |  |  |
| Home mirror training | 0.123 0.312 | 0.119 0.332 | 0.035 0.775 | 0.031 0.798 | 0.228 0.060 | -0.114 0.349 |
| Supportive eye protection | 0.052 0.681 | -0.049 0.695 | 0.042 0.737 | -0.051 0.686 | 0.028 0.821 | 0.012 0.925 |
| Facial training with therapist | 0.022 0.864 | -0.128 0.310 | -0.186 0.139 | -0.111 0.379 | 0.329 **0.007** | -0.241 0.053 |
| Facial training with computer | 0.200 0.113 | 0.270 **0.031** | 0.037 0.772 | 0.156 0.217 | 0.442 **<0.0001** | -0.234 0.063 |
| Biofeedback training | 0.184 0.145 | 0.156 0.217 | -0.062 0.628 | 0.043 0.739 | 0.396 **0.001** | -0.168 0.183 |
| Eye lid surgery | 0.074 0.551 | 0.160 0.195 | 0.083 0.502 | 0.051 0.679 | 0.078 0.530 | 0.153 0.217 |
| Acupuncture | 0.018 0.888 | 0.083 0.506 | 0.018 0.888 | 0.048 0.701 | 0.158 0.202 | -0.020 0.870 |
| Light/heat/cold therapy | 0.121 0.333 | 0.031 0.807 | -0.010 0.937 | -0.171 0.170 | 0.196 0.115 | -0.082 0.513 |
| Electrostimulation | -0.049 0.698 | 0.069 0.586 | 0.178 0.156 | -0.003 0.982 | 0.111 0.381 | -0.028 0.827 |
| Botulinum toxin | 0.072 0.559 | -0.065 0.601 | -0.166 0.175 | -0.009 0.944 | 0.250 **0.039** | -0.181 0.139 |

*p-values <0.05 in bold; **all with the categories: 0=no/1=yes; PROM = Patient-reported outcome measures; FaCE = Facial Clinimetric Evaluation; SF = Short Form; LSAS = Liebowitz Social Anxiety Scale; PHQ = Patient Health Questionnaire; TA = Technology affinity
